# Supplementary material for: Mycobacterium tuberculosis strain with deletions in menT3 and menT4 is attenuated and confers protection in mice and guinea pigs
Source: Nat Commun. 2024 Jun 27;15:5467. doi: 10.1038/s41467-024-49246-5 (PMC11211403; doi:10.1038/s41467-024-49246-5)
Supplement: Supplementary file 1 — Supplementary Information [file 41467_2024_49246_MOESM1_ESM.pdf]

Supplementary information

Supplementary Table 1: List of plasmids, bacterial strains and cell lines used in the study

Plasmids/Strains	Description	Source
pGEM-T easy	TA cloning vector , *amp*^{r}	Promega
pGEM-T easy-*menT3*	pGEM-T easy harboring *menT3*	This study
pGEM-T easy-*menT3*^{G62A}	pGEM-T easy harboring *menT3*^{G62A}	This study
pGEM-T easy-*menT3*^{K189A}	pGEM-T easy harboring *menT3*^{K189A}	This study
pGEM-T easy-*menT3*^{D208A}	pGEM-T easy harboring *menT3*^{D208A}	This study
pGEM-T easy-*menT4*	pGEM-T easy harboring *menT4*	This study
pGEM-T easy-*menT4*^{G51A}	pGEM-T easy harboring *menT4*^{G51A}	This study
pGEM-T easy-*menT4*^{D71A}	pGEM-T easy harboring *menT4*^{D71A}	This study
pET28b	IPTG inducible expression vector	Merck
pET28b-*menT3*	pET28b harboring *menT3*	This study
pET28b-*menT3*^{G62A}	pET28b harboring *menT3*^{G62A}	This study
pET28b-*menT3*^{K189A}	pET28b harboring *menT3*^{K189A}	This study
pET28b-*menT3*^{D208A}	pET28b harboring *menT3*^{D208A}	This study
pET28b-*menT4*	pET28b harboring *menT4*	This study
pET28b-*menT4*^{G51A}	pET28b harboring *menT4*^{G51A}	This study
pET28b-*menT4*^{D71A}	pET28b harboring *menT4*^{D71A}	This study
pETDuet	Modified IPTG inducible vector	Deep et al., 2017
pETDuet-*menT3*	pETDuet harboring *menT3*	This study
pETDuet-*menA2T3*	pETDuet harboring *menA2* and *menT3*	This study
pETDuet-*menA3T3*	pETDuet harboring *menA3* and *menT3*	This study
pETDuet-*menA4T3*	pETDuet harboring *menA4* and *menT3*	This study
pETDuet-*menT4*	pETDuet harboring *menT4*	This study
pETDuet-*menA2T4*	pETDuet harboring *menA2* and *menT4*	This study
pETDuet-*menA3T4*	pETDuet harboring *menA3* and *menT4*	This study
pETDuet-*menA4T4*	pETDuet harboring *menA4* and *menT4*	This study
pTetR	anhydrotetracycline based inducible vector system	Ehrt et al., 2005
pTetR-*menT3*	pTetR harboring *menT3*	This study
pTetR-*menT4*	pTetR harboring *menT4*	This study
pYUB854	Cloning vector, *hyg*^{r}	Bardarov et al., 2002
pYUB854-Δ*menT3 kan*	pYUB854 with *menT3* upstream and downstream region flanking the kanamycin resistance gene	This study

pYUB854-Δ*menT4*	pYUB854 with *menT4* upstream and downstream region flanking the hygromycin resistance gene	This study
pYUB159	Phagemid DNA	Bardarov et al., 2002
pYUB159-Δ*menT3 kan*	pYUB159 derivative to replace *menT3* with kanamycin resistance gene in *M. tuberculosis*	This study
pYUB159-Δ*menT4*	pYUB159 derivative to replace *menT4* with hygromycin resistance gene in *M. tuberculosis*	This study
pMV306 - apramycin	*E. coli mycobacterium* shuttle vector, apramycin	A Kind gift from Dr. Bill Jacobs
pMV306 – *menA3T3*	pMV306 harboring *menA3* and *menT3* locus	This study
pMV306 – *menA4T4*	pMV306 harboring *menA4* and *menT4* locus	This study
E.coli XL-1 Blue	*recA*1 *endA*1 *gyrA*96 *thi*$^{-1}$ *hsdR*17 *supE*44 *relA*1 *lac* [F′ *proAB lacI*q ZΔM15 Tn10 (Tetr)]	Stratagene, USA
E.coli HB-101	F^- , *thi*$^{-1}$, *hsdS*20 (r^{B-} m^B), *supE*44, *recA*13, *ara*$^{-14}$, *leuB*6, *proA*2, *lacY*1, *galK*2, *rpsL*20 (str^r), *xyl*-5, *mtl*-	Promega, USA
BL-21(λDE3) pLysS	*F*$^-$, *ompT*, *hsd*S B (r_B-m_B), *dcm*, *gal*, *λ(DE3), plysS, cm*	Promega, USA
M. smegmatis mc^{2}155	*M. smegmatis* laboratory strain	A kind gift from Dr. Anil K. Tyagi.
M. tuberculosis $H_{37}Rv$	*M. tuberculosis* laboratory strain used to generate overexpression strains and perform protection studies	ATCC
M. tuberculosis Erdman	*M. tuberculosis* laboratory strain used to generate mutant strains.	ATCC
Δ*menT3*	*menT3* mutant strain of *M. tuberculosis* Erdman	This study
Δ*menT4*	*menT4* mutant strain of *M. tuberculosis* Erdman	This study
Δ*menT4*Δ*T3*	*menT3* and *menT4* double mutant strain of *M. tuberculosis* Erdman	This study
Δ*menT4*Δ*T3: menT3*	Δ*menT4*Δ*T3* complemented with *menT3*	This study

Δ*menT4*Δ*T3: menT4*	Δ*menT4*Δ*T3* complemented with *menT4*	This study
THP-1	Human monocyte cell line	NCCS, Pune

Supplementary Table 2: List of primers used in the study

Primer name	Forward primer (5'-3')	Reverse primer (5'-3')
menT3 (pET28b)	GCATATGGTGACCAAGCCCTATTCGTCGCC	GAAGCTTTCATCTTTTCGTCGCCCGATCAA
menT4 (pET28b)	GCATATGGTGGCCGGTCTGACCCGTGCGCT	GAAGCTTTCAGGACCGCAGCACCGCCAGCG
menT3^{G62A} (pET28b)	GTTGGTCAAAGCCGGATCGTCGC	GCGACGATCCGGCTTTGACCAAC
menT3^{K189A} (pET28b)	AATCGCGCAGGCCCTGCACGCAG	CTGCGTGCAGGGCCTGCGCGATT
menT3^{D208A} (pET28b)	CCGCGCTCACGCCCTGGTGGACT	AGTCCACCAGGGCGTGAGCGCGG
menT4^{G51A} (pET28b)	GGTGTTCAAAGCCGGTACCAGCT	AGCTGGTACCGGCTTTGAACACC
menT4^{D71A} (pET28b)	TGATCTGGCCTTCAGCGCGC	GCGCGCTGAAGGCCAGATCA
menA2 (pETDuet)	GCATATGGATCAGATCGGGGCTGACCTCG	GCTCGAGTCATAATCGGGCGAGTCGCTCATCC
menT3 (pETDuet)	GGCTAGCGTGACCAAGCCCTATTCGTCGC	GAAGCTTTCATCTTTTCGTCGCCCGATCAA
menA3 (pETDuet)	GCATATGTTGTGTGCAAAACCGTATCTAATTGAT	GCTCGAGTCACGCCGATGCTCGCTTCGGCCG
menT4 (pETDuet)	GGCTAGCGTGGCCGGTCTGACCCGTGCGCT	GAAGCTTTCAGGACCGCAGCACCGCCAGCG
menA4 (pETDuet)	GCATATGGTGGTGAGCCCAGCCGGCGCCGATC G	GCTCGAGTCACGCCTTGCCGATCACGCGCAGC
menT3-upstream	GGATATCGGTGCTTTCCGCGCCATCCGCCG	GTCTAGACGTTGGCGGCGACGAATAGGGCTTG
menT3-downstream	GAAGCTTGATCGGGCGACGAAAAGATGAGTG	GACTAGTGGCTACGACACGCCCACCGGCC
menT4-upstream	GAGGCCTCCGCCATCCGCGAACTGCGCCG	GTCTAGAGTATGCCTCGGCCCGACCAAGAGC
menT4-downstream	GAAGCTTCGCCTGCGACGAACGGCACCGC	GACTAGTGGCGTCACCGGCAGCTGCACCC
menT3-HR	CTACGCGCTGCGCTCGATGAGACC	GAGGGCGATTCCATTCGTCGTCTG
menT4-HR	CCACCGCGCACAGCCGCGACCGTG	ACCACAGCCGGATTCGTGCCCAAC
menAT3 (pMV306 apra)	GTCTAGAGGGTCCCAACCGAGCGGCAGCAG	GAAGCTTTCATCTTTTCGTCGCCCGATCAA
menAT4 (pMV306 apra)	GTCTAGAGCCCAAGCATCGGCTGGCCGTGCTG	GAAGCTTTCAGGACCGCAGCACCGCCAGCG
**List of primers used for qPCR studies**		
sigA	AGCTGAGGCGGCCGTCGCGCCCGGC	CTACCTTGCCGATCTGTTTGAGGTAGG
menA3	GACGATCTCGCAGCCGCAGTG	CCGTCGACCGAAGTGACGTGGG
menT3	CTTCGACACGGTCGCACGTCGCG	CCGGCTTCGACGGAGGAGACCTC
menA4	CATGCTGGCCGGTGCATCCGCAG	CGAGGTCGAGCCGCCGCCGGAC
menT4	GACGATGAGGTCGTGCTGGAGG	GCGAGCAGCTCAGACGGCAAAGC
Erdman_1164	CAGCGTGGCGGCAACCTCTGGTTGTC	AGACCTGCGCTCGTGCCCGCGAGCCC
Erdman_1167	GGTGCGCGAGGCAGGCCGCGTCGTCG	CGGCCACCAGGCCGGCGTGGGCGTCG
Erdman_3095	AACCCGTACAGTCAGTACGCGCTGGC	ATGCGCGCGAATTGACTTAGCGGGTTC
Erdman_3096	GGTGGCGGCCCACGAGTTCTTGTTGTTC	AGATGTGCAGATCCTCGATGGCGTCC
Erdman_3101	CAGCACCGACGAGTGTGTGCTACTGC	GCCGGACCGGCTCGGCCAGCGGGATCG

Supplementary Table 3: List of various antibodies used for immune studies.

Mouse antibody	Fluorochrome used
CD45.2	APC-Cy7
CD4	PerCp-Cy5.5
CD8	PE, BV510
CD44	PE-Cy7
CD62L	APC
CD69	FITC
IFN-γ	PE
T-bet	APC
IL-17A	PE-Cy7
FoxP3	BV421

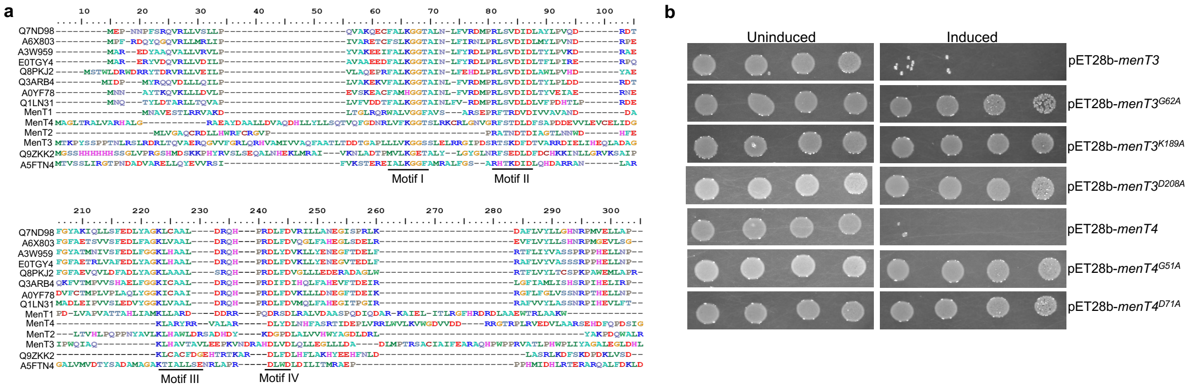

Supplementary Figure 1: (a) Multiple sequence alignment of toxins belonging to type IV TA systems. Multiple sequence alignment of toxins was performed using Clustal W and BioEdit software. The four conserved motifs present in toxins have been highlighted in this panel. The proteins used in alignment are the following; Q7ND98 from *Gleobacter violaceous*, A6X803 from *Brucella anthropi*, A3W959 from *Roseovarius sp. 217*, E0TGY4 from *Parvularcula bermudensis*, Q8PKJ2 from *Xanthomonas axonopodis*, Q3ARB4 from *Chlorobium chlorochromatii*, A0YF78 from *Marine gamma proteobacterium HTCC2143*, Q1LN31 from *Cupriavidus metallidurans*, MenT1, Rv0078a from *M. tuberculosis*, MenT4, Rv2826c from *M. tuberculosis*, MenT2, Rv0836c from *M. tuberculosis*, MenT3, Rv1045 from *M. tuberculosis,* Q9ZKK2 from *Helicobacter pylori* and A5FTN4 from *Acidiphilium cryptum*. **(b) Ectopic expression of MenT3 and MenT4 inhibits *E. coli* growth.** *E. coli* BL-21 (λDE3, plysS) harbouring various pET28b constructs were cultured in LB medium till OD_{600nm} ~ 0.4-0.6. The expression of wild type and mutant toxins was induced by the addition of 1.0 mM IPTG. For spotting assays, after 4 h of induction, cultures were serially diluted and spotted on LB agar plates. The data shown in b is representative of two independent experiments.

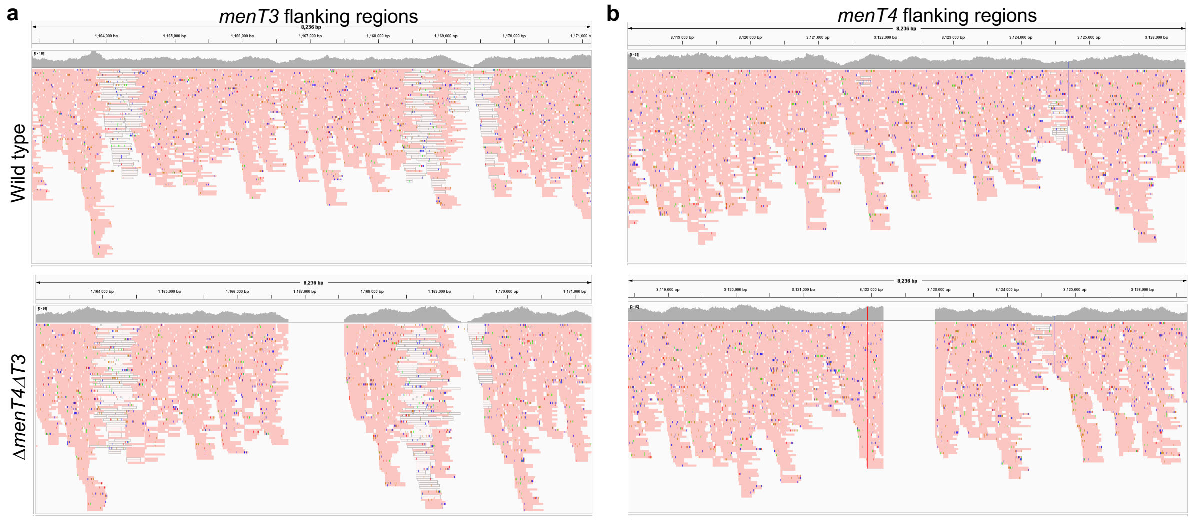

Supplementary Figure 2: Construction of Δ*menT4*Δ*T3* strain of *M. tuberculosis*. The replacement of *menT3* and *menT4* with kanamycin and hygromycin resistance genes, respectively, in Δ*menT4*Δ*T3* strain of *M. tuberculosis* was confirmed by whole genome sequencing. The sequencing reads around *menT3* (a) and *menT4* (b) flanking regions obtained from wild type and Δ*menT4*Δ*T3* were aligned on the reference genome sequence of *M. tuberculosis* Erdman.

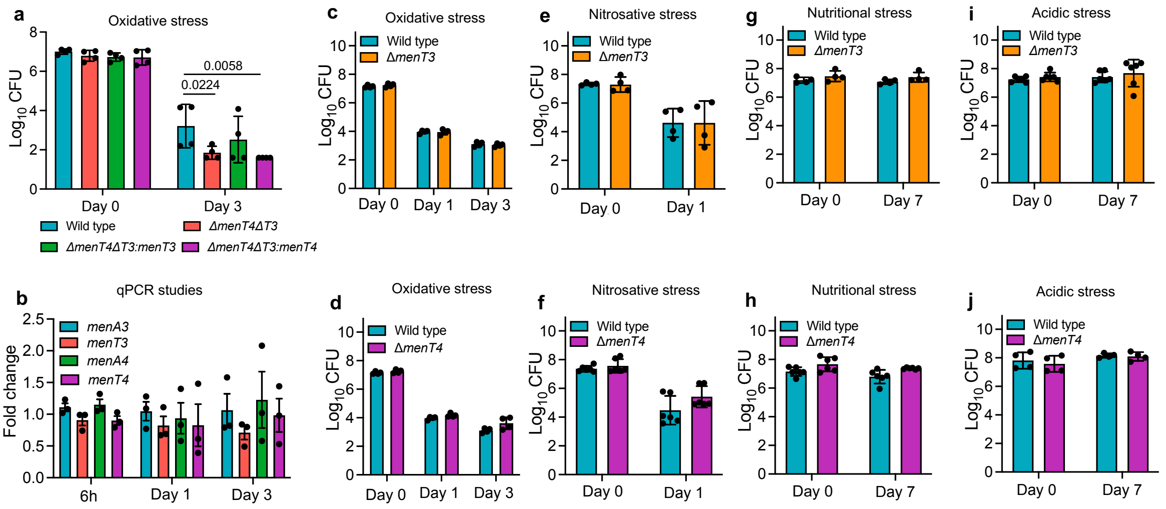

Supplementary Figure 3: (a) MenT3 and MenT4 are essential for *M. tuberculosis* survival in oxidative stress conditions. The data shown in this panel is mean $\pm$ SD of $\log_{10}$ CFU of various strains before and after exposure to oxidative stress for 3 days. The data is obtained from two independent experiments, each performed with duplicate cultures. *P-values* depicted on the graphs were assessed using 2way ANOVA. **(b) qRT-PCR analysis of *menA3*, *menT3*, *menA4*, *menT4* upon exposure to oxidative stress conditions.** Early-log phase culture of *M. tuberculosis* was exposed to oxidative stress for either 6 h or 24 h or 72 h. qRT-PCR was performed, and the data obtained was normalized to the levels of *sigA*. The data represents mean $\pm$ SEM obtained from three independent experiments. **(c-j) Susceptibility of wild type, Δ*menT3* and Δ*menT4* to oxidative, nitrosative, nutritional and acidic stress.** Various strains were grown till $OD_{600nm} \sim$ 0.2 as described in materials and methods. The data shown in these panels is mean $\pm$ SD of $\log_{10}$ CFU of various strains before and after exposure to various stress conditions from two (c, d, e, g, j) or three (f, h, i) independent experiments, each performed with duplicate cultures. The CFU values for wild type samples in panels (c) and (d) are similar. Source Data are provided as a Source Data file.

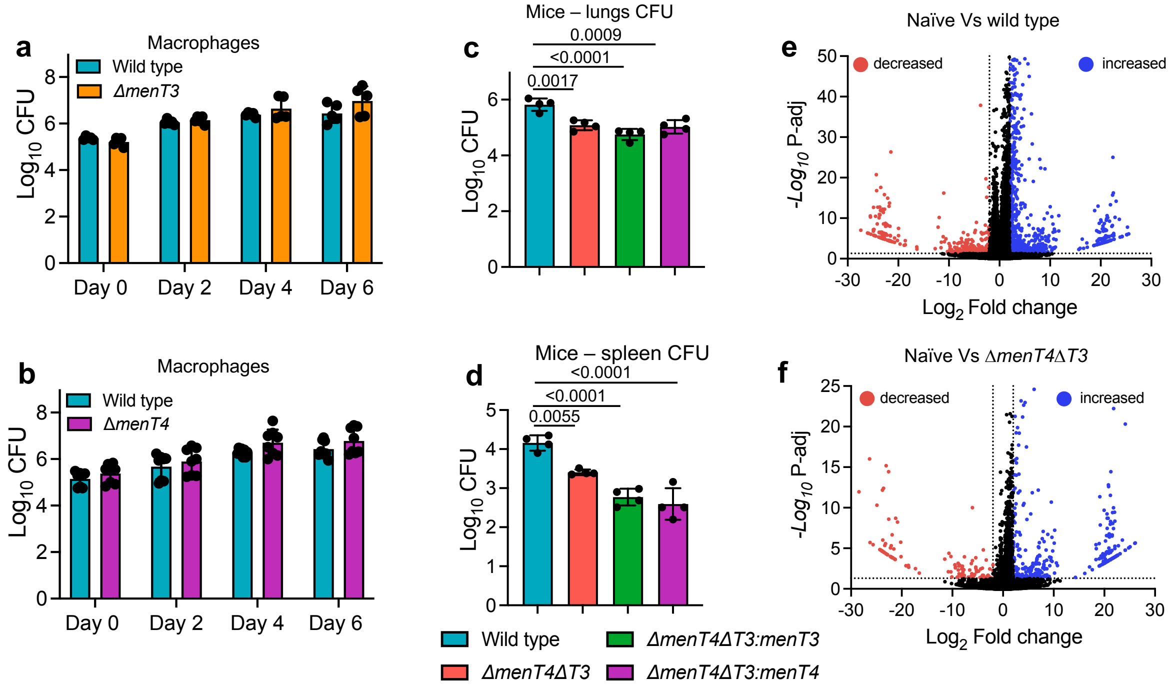

Supplementary Figure 4: (a - b) Effect of deletion of *menT3* or *menT4* on the intracellular growth of *M. tuberculosis*. THP-1 macrophages were infected with various strains, and bacterial enumeration was performed as described in the materials and methods. The data shown in these panels is mean ± SD of $\log_{10}$ CFU obtained from two (a) or three (b) independent experiments, each performed in duplicates or triplicates. **(c - d) Deletion of *menT3* and *menT4* impairs *M. tuberculosis* growth in the lungs and spleens of mice.** The bacterial loads were determined in lungs (c) and spleens (d) of mice infected with various strains at 4 weeks post-infection. The data shown in this panel is mean ± SD of $\log_{10}$ CFU obtained from 4 animals per group. The data shown for wild type and Δ*menT4*Δ*T3* is representative of two experiments. The data shown for the single complemented strain is obtained from a single experiment. *P-values* depicted on the graphs were assessed using one-way ANOVA. **(e - f) Host transcriptional profiles of lung tissues from uninfected or mice infected with either wild type or Δ*menT4*Δ*T3* strain at 4 weeks post-infection.** Volcano plot showing comparative transcriptional profiles obtained from uninfected lung tissues with mice infected with either wild type (e) or Δ*menT4*Δ*T3* (f) strain. The transcripts with increased or decreased expression in mice infected with parental (e) or Δ*menT4*Δ*T3* (f) strain in comparison to uninfected animals have been shown as blue and red dots, respectively. Black dots represent the transcripts that were not differentially expressed between these two groups. The data shown in panels e and f is obtained from three independent biological replicates. Source Data are provided as a Source Data file.

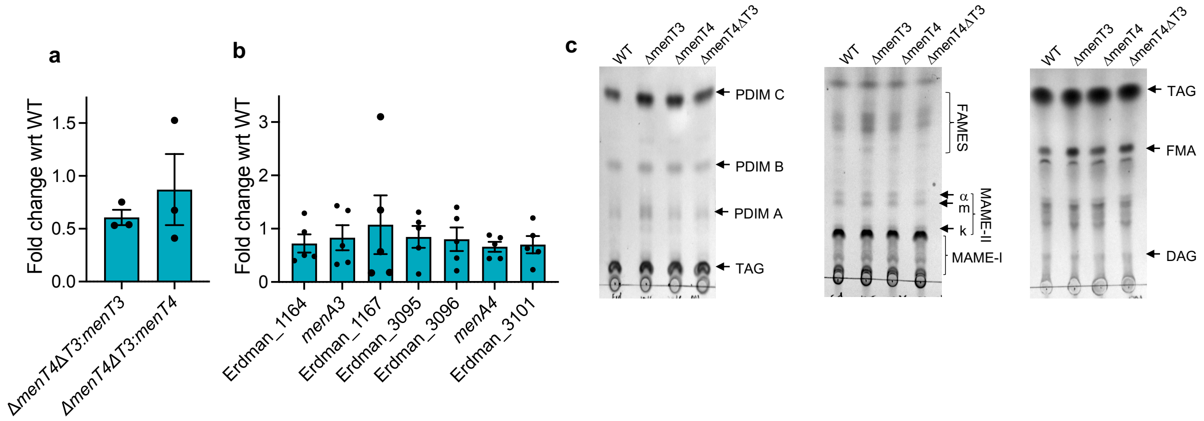

Supplementary Figure 5: (a) Relative expression analysis of *menT3* and *menT4* in single complemented strains. For qRT-PCR analysis, wild type, Δ*menT4*Δ*T3*: *menT3* and Δ*menT4*Δ*T3*: *menT4* were grown till the mid-log phase (OD_{600nm}~ 0.8-1.0), total mRNA was isolated, and qRT-PCR was performed using gene-specific primers. The data shown in this panel is mean ± SEM of fold change obtained in complemented strains relative to the parental strain obtained from three independent experiments. **(b) Relative expression analysis of *menT3* and *menT4* neighbouring genes in wild type and Δ*menT4*Δ*T3* strains.** The data shown in this panel is mean ± SEM of fold change obtained in mid-log phase cultures of Δ*menT4*Δ*T3* relative to the parental strain obtained from five independent experiments. **(c) Lipids profile of the wild type, Δ*menT3*, Δ*menT4* and Δ*menT4*Δ*T3* strains of *M. tuberculosis*.** Apolar and polar total lipid fractions were isolated from mid-log phase cultures (OD_{600nm} ~ 0.8-1.0) of various strains. An Equal amount of different lipid fractions were spotted on silica plates and resolved using one dimensional-TLC in different solvent systems. The TLC plates were visualized using 5 % molybdophosphoric acid in ethanol followed by charring at 100 °C. The TLC images were acquired using a gel documentation system (Bio-Rad). TAG-triacylglycerol, PDIM-phthiocerol dimycocerosate, FAMES-fatty acid methyl esters and MAME-mycolic acid methyl esters, DAG- diacylglycerol, FMA- free mycolic acid. The data shown in panel c is from a single experiment. Source Data are provided as a Source Data file.

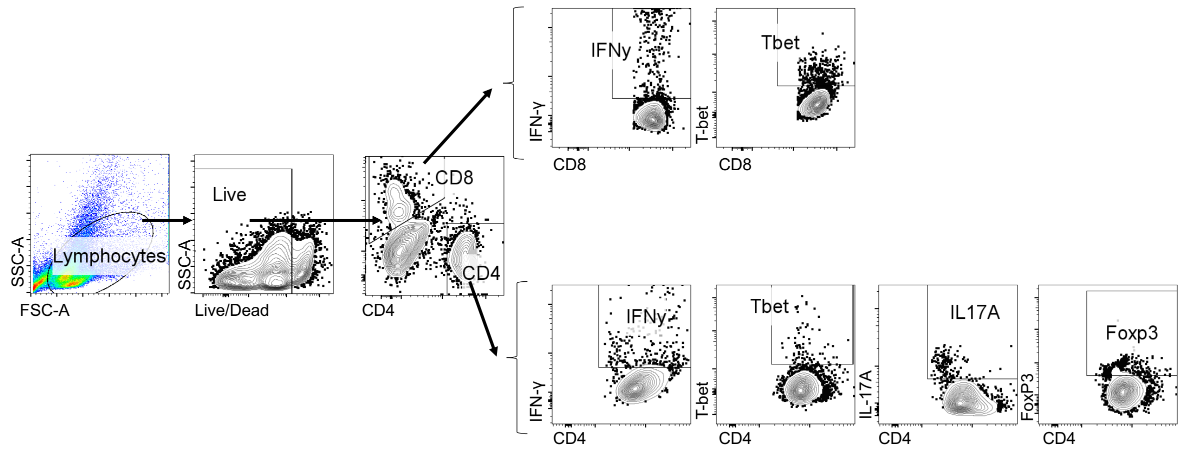

Supplementary Figure 6: Gating strategy for the measurement of intracellular cytokines in spleens of naive or BCG or Δ*menT4*Δ*T3* immunized mice. Single-cell suspension was prepared from spleen harvested from mice at 6 weeks post-immunization. The cells were seeded in a 96 well plate and stimulated with PPD for 72 h to determine intracellular cytokine levels. Subsequently, cells were stained with surface and intracellular cytokines as described in materials and methods. The data was acquired on BD Canto and analyzed using FlowJo software. The gating strategy was followed in a sequential manner (left to right), as shown in the FACS plots above. IFN-γ^+, T-bet$^+$, IL-17A$^+$ and Foxp3$^+$ cells were gated on CD4$^+$ T cells or CD8$^+$ T cells as indicated above.

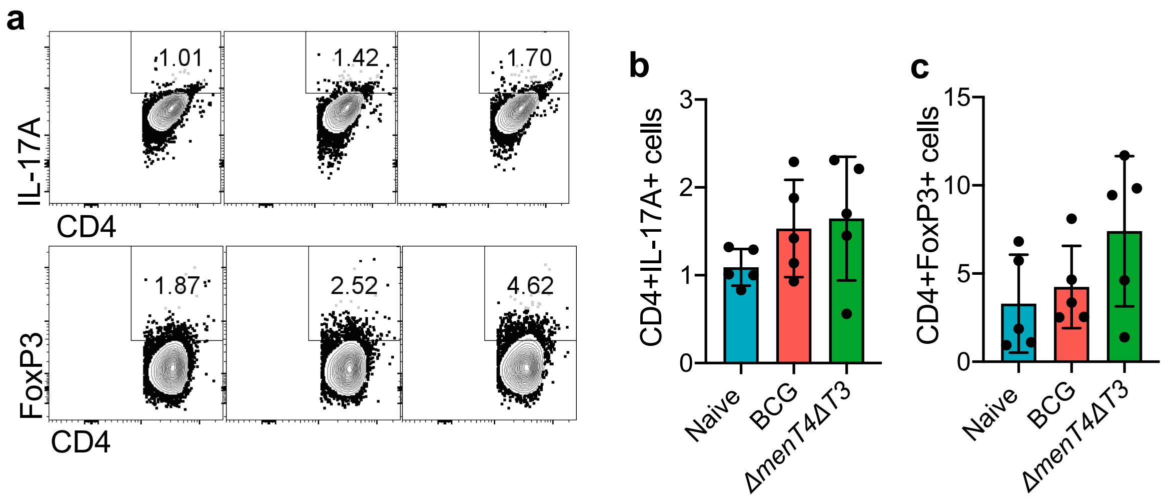

Supplementary Figure 7: Evaluation of IL-17A and FoxP3 positive CD4$^+$ T cells in naive or BCG or Δ*menT4*Δ*T3* immunized mice. (a) Representative FACS plots showing frequency of CD4$^+$ T-cells positive IL-17A$^+$ and FoxP3$^+$ in spleens of naive or BCG or Δ*menT4*Δ*T3* immunized mice. **(b - c)** Bar graph showing percentage frequency mean $\pm$ SD of CD4$^+$ IL-17A$^+$ T cells (b) and CD4$^+$FoxP3$^+$ T cells (c) is obtained from 5 animals from a single experiment. Source Data are provided as a Source Data file.

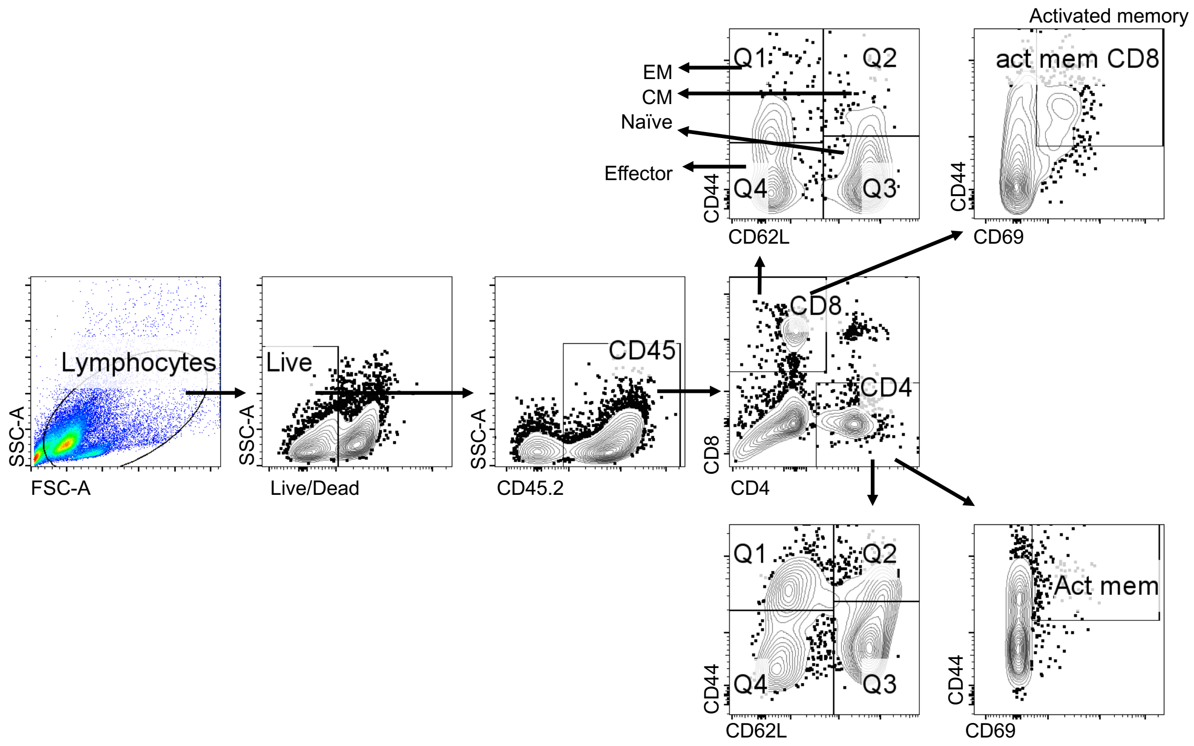

Supplementary Figure 8: Gating strategy for the measurement of memory T-cell responses in naive or BCG or Δ*menT4*Δ*T3* immunized mice. Single cell suspension was prepared from spleens of immunized mice at 6 weeks post-immunization. The cells were seeded in a 96 well plate and surface stained as described in materials and methods. The data was acquired on BD Canto and analyzed using FlowJo software. The gating strategy was followed in a sequential manner (left to right) as shown in the FACS plots above. Effector memory (EM), central memory (CM), Effector and naïve T cells, along with activated memory T cells were gated on $CD4^+$ or $CD8^+$ T cells as indicated above.

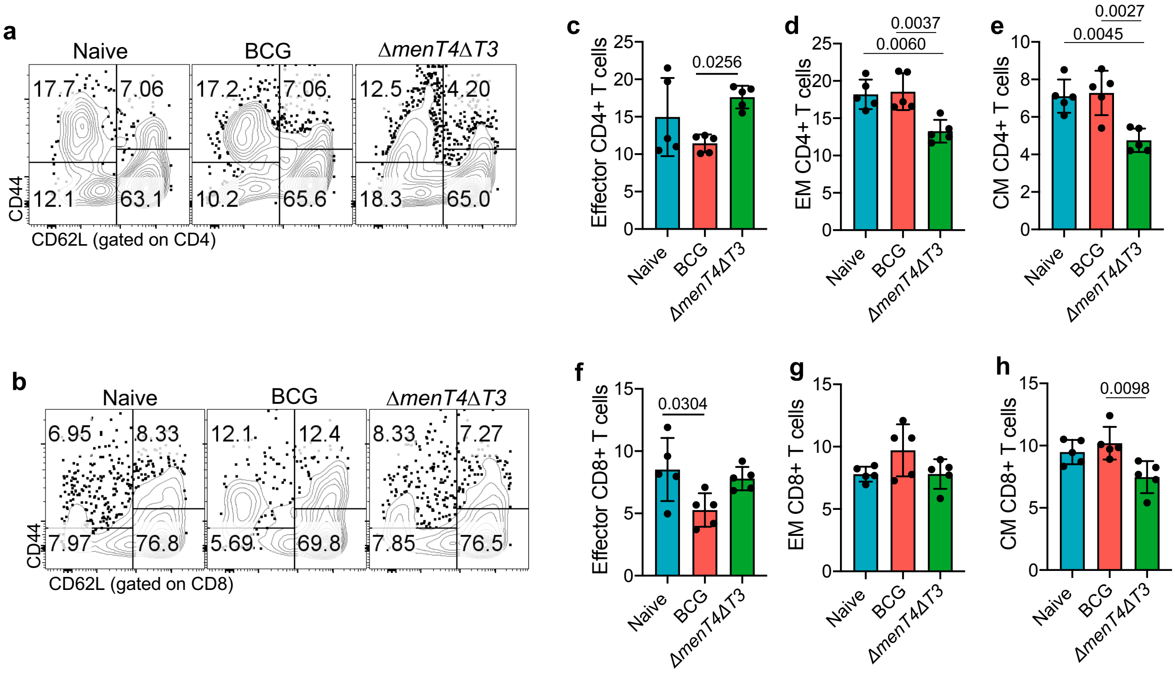

Supplementary Figure 9: Evaluation of memory T cell response in naive or BCG or Δ*menT*4Δ*T*3 immunized mice. (a and b) Representative FACS plots showing the frequency of effector, effector memory and central memory $CD4^+$ T_H cells (a) and $CD8^+$ T_C cells (b) in spleens of naive or BCG or Δ*menT4*Δ*T3* immunized mice. (c-e, f-h) These panels show the proportion of effector $CD4^+$ T_H cells (c) effector memory $CD4^+$ T_H cells (d), central memory $CD4^+$ T_H cells (e), effector $CD8^+$ T_C cells (f), effector memory $CD8^+$ T_C cells (g) and central memory $CD8^+$ T_C cells (h) in spleens of naive, BCG and Δ*menT4*Δ*T3* immunized mice. The data shown in these panels is mean $\pm$ SD of the proportion of cells obtained from 5 animals from a single experiment. *P-values* depicted on the graphs were assessed using one-way ANOVA. Source Data are provided as a Source Data file.

Raw images for spotting assays showed in Figure S1b.

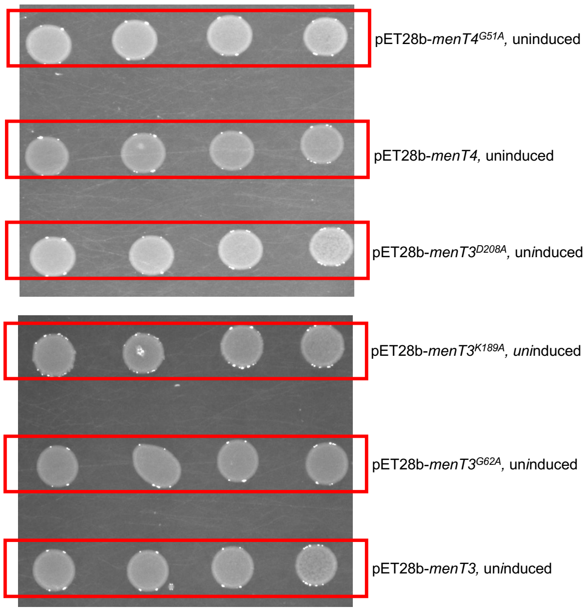

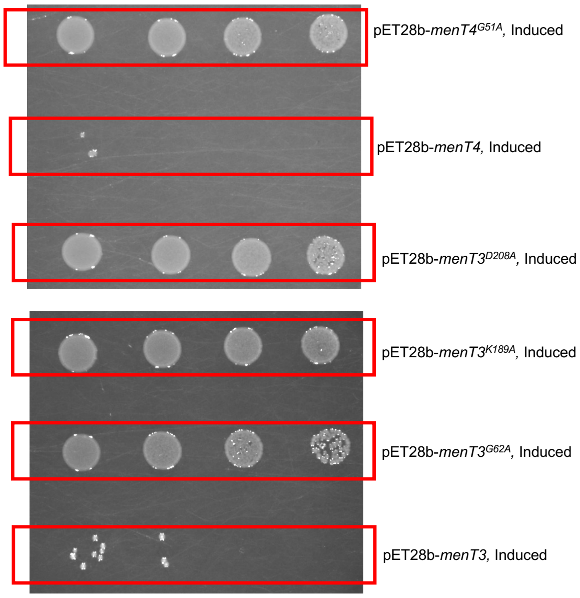

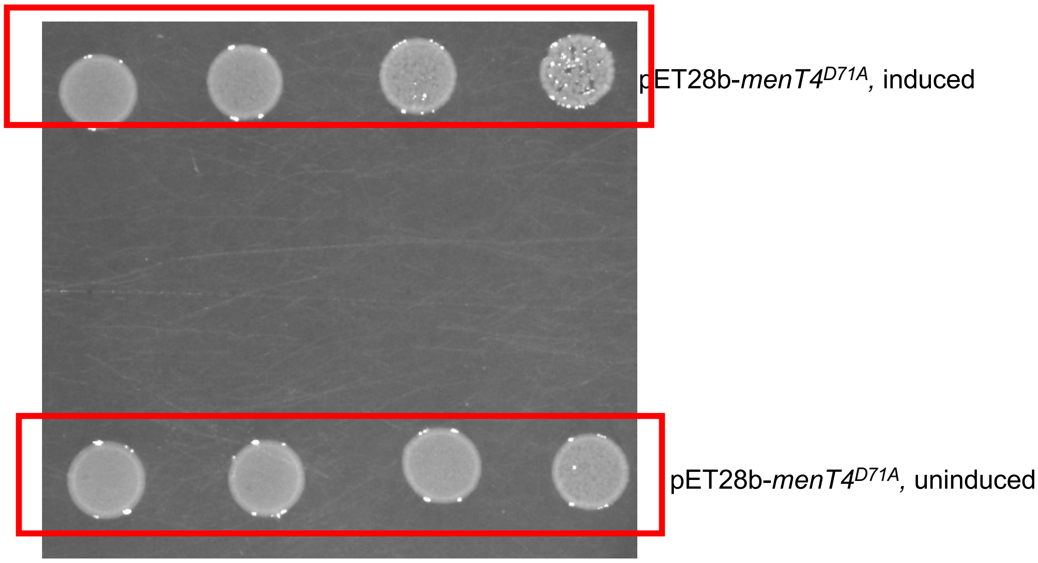

Raw images for TLC plates shown in Fig. S5c.

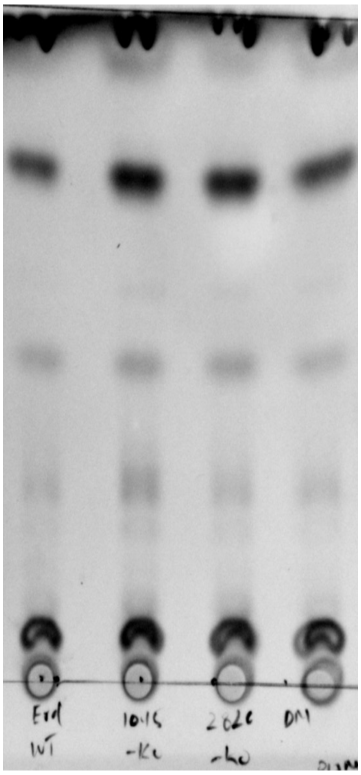

PDIM raw image

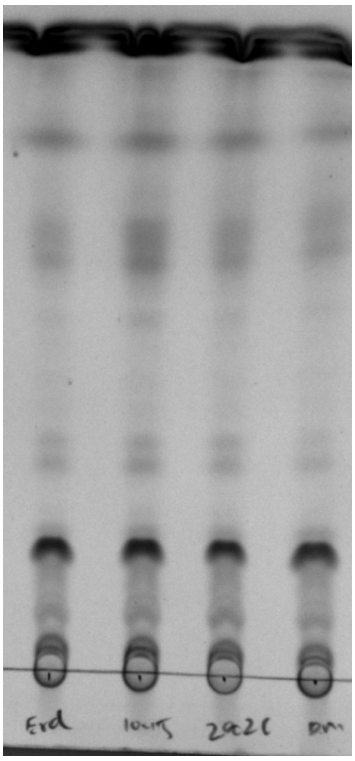

Mycolic acid raw image

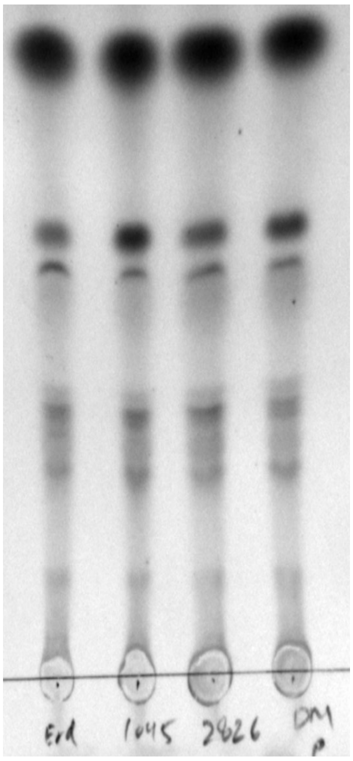

Polar lipid raw image
