# Supplementary material for: Mycobacterium tuberculosis strain with deletions in menT3 and menT4 is attenuated and confers protection in mice and guinea pigs
Source: Nat Commun. 2024 Jun 27;15:5467. doi: 10.1038/s41467-024-49246-5 (PMC11211403; doi:10.1038/s41467-024-49246-5)
Supplement: Supplementary file 3 — Description of Additional Supplementary Files [file 41467_2024_49246_MOESM3_ESM.pdf]

Legends to Supplementary Data Files

Supplementary Data 1: List of differentially expressed genes in mid-log phase cultures of Δ*menT4*Δ*T3* mutant strain *M. tuberculosis* in comparison to the wild type strain using cutoff values of 2.0-fold change and *P value* of ≤ 0.05.

Supplementary Data 2: List of differentially expressed genes in lungs of *M. tuberculosis* Erdman infected mice with respect to Naïve mice using a fold change cutoff of $\log_2$ fold change of +2, -2 and *Padj value* of ≤ 0.05.

Supplementary Data 3: List of differentially expressed genes in lungs of Δ*menT4*Δ*T3* infected mice with respect to Naïve mice using a fold change cutoff of $\log_2$ fold change of +2, -2 and *Padj value* of ≤ 0.05.

Supplementary Data 4: List of differentially expressed genes in lungs of Δ*menT4*Δ*T3* infected mice with respect to *M. tuberculosis* Erdman infected mice using a fold change cutoff of log2 fold change of +2, -2 and *Padj value* of ≤ 0.05.
