# Supplementary material for: Mycobacterium tuberculosis strain with deletions in menT3 and menT4 is attenuated and confers protection in mice and guinea pigs
Source: Nat Commun. 2024 Jun 27;15:5467. doi: 10.1038/s41467-024-49246-5 (PMC11211403; doi:10.1038/s41467-024-49246-5)
Supplement: Supplementary file 8 — Reporting Summary [file 41467_2024_49246_MOESM8_ESM.pdf]

nature portfolio

Corresponding author(s): Ramandeep Singh

Last updated by author(s): 06/05/24

Reporting Summary

Nature Portfolio wishes to improve the reproducibility of the work that we publish. This form provides structure for consistency and transparency in reporting. For further information on Nature Portfolio policies, see our Editorial Policies and the Editorial Policy Checklist.

Please do not complete any field with "not applicable" or n/a. Refer to the help text for what text to use if an item is not relevant to your study.
For final submission: please carefully check your responses for accuracy; you will not be able to make changes later.

Statistics

For all statistical analyses, confirm that the following items are present in the figure legend, table legend, main text, or Methods section.

n/a	Confirmed	
☐	☑	The exact sample size (*n*) for each experimental group/condition, given as a discrete number and unit of measurement
☐	☑	A statement on whether measurements were taken from distinct samples or whether the same sample was measured repeatedly
☐	☑	The statistical test(s) used AND whether they are one- or two-sided<br>*Only common tests should be described solely by name; describe more complex techniques in the Methods section.*
☑	☐	A description of all covariates tested
☑	☐	A description of any assumptions or corrections, such as tests of normality and adjustment for multiple comparisons
☐	☑	A full description of the statistical parameters including central tendency (e.g. means) or other basic estimates (e.g. regression coefficient) AND variation (e.g. standard deviation) or associated estimates of uncertainty (e.g. confidence intervals)
☑	☐	For null hypothesis testing, the test statistic (e.g. *F*, *t*, *r*) with confidence intervals, effect sizes, degrees of freedom and *P* value noted<br>*Give P values as exact values whenever suitable.*
☑	☐	For Bayesian analysis, information on the choice of priors and Markov chain Monte Carlo settings
☑	☐	For hierarchical and complex designs, identification of the appropriate level for tests and full reporting of outcomes
☑	☐	Estimates of effect sizes (e.g. Cohen's *d*, Pearson's *r*), indicating how they were calculated

Our web collection on statistics for biologists contains articles on many of the points above.

Software and code

Policy information about availability of computer code

Data collection: Bacterial RNA sequencing was performed using the Illumina HiSeq2000 Platform, and host RNA sequencing was performed using the Illumina Novaseq6000.

Data analysis: Raw data for experiments was analysed using Microsoft Excel and GraphPad Prism (version 9.5.1). All statistical analysis was performed using GraphPad Prism (version 9.5.1). Differential gene expression analysis of the bacterial RNA sequencing data was performed using the Cuffdiff program of the cufflinks package. Differential gene expression analysis of the host RNA sequencing data was performed using DeSeq2. Flow cytometry data was analysed using FlowJo (Treestar) software version X.

For manuscripts utilizing custom algorithms or software that are central to the research but not yet described in published literature, software must be made available to editors and reviewers. We strongly encourage code deposition in a community repository (e.g. GitHub). See the Nature Portfolio guidelines for submitting code & software for further information.

Data

Policy information about availability of data

All manuscripts must include a data availability statement. This statement should provide the following information, where applicable:

- Accession codes, unique identifiers, or web links for publicly available datasets
- A description of any restrictions on data availability
- For clinical datasets or third party data, please ensure that the statement adheres to our policy

The raw data generated during the study has been provided as a separate MS Excel spreadsheet labelled "Source Data file". The RNA-seq data generated in the study have been deposited in NCBI-SRA repositories under accession code Bioproject PRJNA997775 (https://www.ncbi.nlm.nih.gov/bioproject/?term=PRJNA997775) for *Mus musculus* and PRJNA997818 (https://www.ncbi.nlm.nih.gov/bioproject/?term=PRJNA997818) for *M. tuberculosis*. The bacterial and host RNA sequencing data set has been provided as Supplementary Data Files.

Research involving human participants, their data, or biological material

Policy information about studies with human participants or human data. See also policy information about sex, gender (identity/presentation), and sexual orientation and race, ethnicity and racism.

Reporting on sex and gender	Not applicable
Reporting on race, ethnicity, or other socially relevant groupings	Not applicable
Population characteristics	Not applicable
Recruitment	Not applicable
Ethics oversight	Not applicable

Note that full information on the approval of the study protocol must also be provided in the manuscript.

Field-specific reporting

Please select the one below that is the best fit for your research. If you are not sure, read the appropriate sections before making your selection.

☑ Life sciences ☐ Behavioural & social sciences ☐ Ecological, evolutionary & environmental sciences

For a reference copy of the document with all sections, see nature.com/documents/nr-reporting-summary-flat.pdf

Life sciences study design

All studies must disclose on these points even when the disclosure is negative.

Sample size	No sample size calculations were made, however, we determined the sample size to be used for various experiments based on studies published in the field from ours and other laboratories. The sample size for various experiments was sufficient to determine mean +/- SD and to perform statistical analysis using two-tailed paired t-test or one-way ANOVA or 2way ANOVA.
Data exclusion	No data points were excluded. For animal experiments, contamination during tissue processing or plating resulted in decreased animal size occasionally.
Replication	Generally, we performed at least two independent experiments with duplicate cultures for each *in vitro* CFU experiment. The details are specified in the respective figure legends. The number of animals used in mice and guinea pig virulence or efficacy studies is also mentioned in the figure legends of the manuscript.
Randomization	For virulence studies, mice and guinea pigs were randomly chosen to be infected with either wild type or mutant strain. For efficacy studies, mice and guinea pigs were randomly chosen to be immunized with BCG or mutant strain before infection with *M. tuberculosis* via aerosol route.
Blinding	Blinding was not generally performed during data collection and analysis. The data was collected, analysed, and validated by different experimenters who independently performed the experiments. The histopathology samples from guinea pig virulence and efficacy studies were coded before being sent to the histopathologist for calculation of total granuloma score.

Behavioural & social sciences study design

All studies must disclose on these points even when the disclosure is negative.

Study description	Not applicable
Research sample	Not applicable
Sampling strategy	Not applicable
Data collection	Not applicable
Timing	Not applicable
Data exclusions	Not applicable
Non-participation	Not applicable
Randomization	Not applicable

Ecological, evolutionary & environmental sciences study design

All studies must disclose on these points even when the disclosure is negative.

Study description	Not applicable
Research sample	Not applicable
Sampling strategy	Not applicable
Data collection	Not applicable
Timing and spatial scale	Not applicable
Data exclusions	Not applicable
Reproducibility	Not applicable
Randomization	Not applicable
Blinding	Not applicable

Did the study involve field work? ☐ Yes ☑ No

Field work, collection and transport

Field conditions	Not applicable
Location	Not applicable
Access & import/export	Not applicable
Disturbance	Not applicable

Reporting for specific materials, systems and methods

We require information from authors about some types of materials, experimental systems and methods used in many studies. Here, indicate whether each material, system or method listed is relevant to your study. If you are not sure if a list item applies to your research, read the appropriate section before selecting a response.

Materials & experimental systems

n/a	Involved in the study	
☐	☑	Antibodies
☐	☑	Eukaryotic cell lines
☑	☐	Palaeontology and archaeology
☐	☑	Animals and other organisms
☑	☐	Clinical data
☑	☐	Dual use research of concern
☑	☐	Plants

Methods

n/a	Involved in the study	
☑	☐	ChIP-seq
☐	☑	Flow cytometry
☑	☐	MRI-based neuroimaging

Antibodies

Antibodies used

A list of antibodies used in the manuscript is provided in Table S3.

Antibody	Fluorochrome	Clone	Dilution	Catalog no.	Manufacturer
CD45.2	APC-Cy7	104	1:600	109823	Biolegend
CD8	PE	53-6.7	1:1000	100753	Biolegend
CD44	PE-Cy7	IM7	1:500	103029	Biolegend
CD62L	APC	MEL-14	1:600	104411	Biolegend
CD69	FITC	H1.2F3	1:600	104505	Biolegend
IFN-γ	PE	XMG1.2	1:400	505807	Biolegend
T-bet	APC	4B10	1:400	644813	Biolegend
CD4	PerCp-Cy5.5	RM4-4	1:1000	100538	Biolegend
CD8	BV510	53-6.7	1:1000	100751	Biolegend
IL-17A	PE-Cy7	TC11-18H10.1	1:400	506922	Biolegend
FoxP3	BV421	MF-14	1:400	126419	Biolegend

Validation

All antibodies were validated by the supplier (Bio Legend) and checked in the lab by comparing manufacturer or in-house results. Statement from Bio legend: Bio legend Antibodies undergo an extensive series of testing to ensure quality at every step in the manufacturing process, as well as maintaining quality after the sale.

The validation statement and the relevant citation information is listed in the link:

1. anti-mouse CD4-Percp cy5.5 (Cat no-100751, Clone-53-6.7, Biolegend INC, USA, 1:1000) https://www.biolegend.com/en-us/products/percp-cyanine5-5-anti-mouse-cd4-antibody-4230?GroupID=BLG4211

2. anti-mouse-CD8 – BV510 (Cat no-100753, Clone-53-6.7 Biolegend INC, USA,1:1000) https://www.biolegend.com/en-us/products/brilliant-violet-510-anti-mouse-cd8a-antibody-7992

3. anti-mouse-CD8 – PE (Cat no-100707, Clone-53-6.7 Biolegend INC, USA,1:1000) https://www.biolegend.com/en-us/products/pe-anti-mouse-cd8a-antibody-155

4. anti-mouse CD45.2 APC-Cy7 (Cat no- 109823, Clone-104, Biolegend INC, USA, 1:600) https://www.biolegend.com/en-us/products/apc-cyanine7-anti-mouse-cd45-2-antibody-3906

5. anti-mouse CD44 PE-Cy7 (Cat no- 103029, Clone-IM7, Biolegend INC, USA, 1:500) https://www.biolegend.com/en-us/products/pe-cyanine7-anti-mouse-human-cd44-antibody-3932

6. anti-mouse CD62L APC (Cat no- 104411, Clone-MEL-14, Biolegend INC, USA, 1:600) https://www.biolegend.com/en-us/products/apc-anti-mouse-cd62l-antibody-381

7. anti-mouse IFNy PE (Cat no- 505807, Clone-XMG1.2, Biolegend INC, USA, 1:400) https://www.biolegend.com/en-us/products/pe-anti-mouse-ifn-gamma-antibody-997

8. anti-mouse CD69 FITC (Cat no- 104505, Clone- H1.2F3, Biolegend INC, USA, 1:400) https://www.biolegend.com/en-us/products/fitc-anti-mouse-cd69-antibody-264

9. anti-mouse T-bet APC (Cat no- 644813, Clone-4B10, Biolegend INC, USA, 1:600) https://www.biolegend.com/en-us/products/apc-anti-t-bet-antibody-7120

10. anti-mouse IL17A PE-Cy7 (Cat no- 506922, Clone- TC11-18H10.1, Biolegend INC, USA, 1:400) https://www.biolegend.com/en-us/products/pe-cyanine7-anti-mouse-il-17a-antibody-6013

11. anti-mouse Foxp3 BV421 (Cat no- 126419, Clone- MF-14, Biolegend INC, USA, 1:400) https://www.biolegend.com/en-us/products/brilliant-violet-421-anti-mouse-foxp3-antibody-12143

Eukaryotic cell lines

Policy information about cell lines and Sex and Gender in Research

Cell line source(s)	THP-1 cell line was purchased from NCSS, Pune
Authentication	The cell line was not authenticated by us.
Mycoplasma contamination	Mycoplasma contamination was ruled out by PCR on a regular basis.
Commonly misidentified lines (See ICLAC register)	No misidentified cell line was used in the study.

Palaeontology and Archaeology

Specimen provenance	Not applicable
Specimen deposition	Not applicable
Dating methods	Not applicable

☐ Tick this box to confirm that the raw and calibrated dates are available in the paper or in Supplementary Information.

Ethics oversight	Not applicable

Note that full information on the approval of the study protocol must also be provided in the manuscript.

Animals and other research organisms

Policy information about studies involving animals; ARRIVE guidelines recommended for reporting animal research, and Sex and Gender in Research

Laboratory animals	6-8 weeks old outbred female Duncan Hartley guinea pigs (~250-300 g) were obtained from Disease Free Small Animal House, Lala Lajpat Rai University of Veterinary and Animal Sciences, Hisar. 6-8 weeks old inbred female Balb/c and C57BL/6 mice (~ 20-25 g) were obtained from the Experimental Animal Facility, NCR Biotech Science Cluster, Faridabad. Animals were housed in a group of either 5 (mice) or 3 (guinea pigs) in individually ventilated cages in BSL-3 labs. The animals were maintained at a room temperature of 22 $\pm$ 3 ^{o}C, relative humidity of 30 to 70 %, 15-20 air changes/hours, light intensity of 325-350 lux with a 14 h light/10 h dark cycle and noise intensity of <85 db.
Wild animals	This study did not involve wild animals.
Reporting on sex	Female mice and guinea pigs were only used in this study.
Field-collected samples	This study did not contain samples collected from the field.
Ethics oversight	The institutional animal ethics committee of the Translational Health Science and Technology Institute (THSTI) approved the animal experiments. The animal experiments were performed as per the guidelines provided by the committee for the control and supervision of experiments on animals.

Note that full information on the approval of the study protocol must also be provided in the manuscript.

Clinical data

Policy information about clinical studies

All manuscripts should comply with the ICMJE guidelines for publication of clinical research and a completed CONSORT checklist must be included with all submissions.

Clinical trial registration	Not applicable
Study protocol	Not applicable
Data collection	Not applicable
Outcomes	Not applicable

Dual use research of concern

Policy information about dual use research of concern

Hazards

Could the accidental, deliberate or reckless misuse of agents or technologies generated in the work, or the application of information presented in the manuscript, pose a threat to:

No	Yes	
☑	☐	Public health
☑	☐	National security
☑	☐	Crops and/or livestock
☑	☐	Ecosystems
☑	☐	Any other significant area

Experiments of concern

Does the work involve any of these experiments of concern:

No	Yes	
☑	☐	Demonstrate how to render a vaccine ineffective
☑	☐	Confer resistance to therapeutically useful antibiotics or antiviral agents
☑	☐	Enhance the virulence of a pathogen or render a nonpathogen virulent
☑	☐	Increase transmissibility of a pathogen
☑	☐	Alter the host range of a pathogen
☑	☐	Enable evasion of diagnostic/detection modalities
☑	☐	Enable the weaponization of a biological agent or toxin
☑	☐	Any other potentially harmful combination of experiments and agents

Plants

Seed stocks	Not applicable
Novel plant genotypes	Not applicable
Authentication	Not applicable

ChIP-seq Not applicable

Data deposition

☐ Confirm that both raw and final processed data have been deposited in a public database such as GEO.

☐ Confirm that you have deposited or provided access to graph files (e.g. BED files) for the called peaks.

Data access links<br>*May remain private before publication.*	Not applicable
Files in database submission	Not applicable
Genome browser session<br>(e.g. UCSC)	Not applicable

Methodology

Replicates	Not applicable
Sequencing depth	Not applicable
Antibodies	Not applicable
Peak calling parameters	Not applicable
	Not applicable

Data quality

Software | Not applicable

Flow Cytometry

Plots

Confirm that:

☑ The axis labels state the marker and fluorochrome used (e.g. CD4-FITC). Fluorochromes and markers are mentioned in Table S2

☑ The axis scales are clearly visible. Include numbers along axes only for bottom left plot of group (a 'group' is an analysis of identical markers).

☒ All plots are contour plots with outliers or pseudocolor plots.

☑ A numerical value for number of cells or percentage (with statistics) is provided.

Methodology

Sample preparation: Spleens were aseptically harvested from the mice, and a single-cell suspension of the spleen was prepared using a 70-micron cell strainer. 0.2 million cells were used for staining with different fluorescently tagged antibodies to identify different cell population.

Instrument: Flow cytometry data were collected on BD FACS Canto II

Software: Flow cytometry data was analysed using FlowJo (Treestar) software version X.

Cell population abundance: Spleen (0.2 million) was used for surface, intra-cellular cytokine staining to identify the different cell populations

Gating strategy: Provided in manuscript

☑ Tick this box to confirm that a figure exemplifying the gating strategy is provided in the Supplementary Information.

Magnetic resonance imaging

Experimental design

Design type: Not applicable

Design specifications: Not applicable

Behavioral performance measures: Not applicable

Imaging type(s): Not applicable

Field strength: Not applicable

Sequence & imaging parameters: Not applicable

Area of acquisition: Not applicable

Diffusion MRI ☐ Used ☑ Not used

Preprocessing

Preprocessing software: Not applicable

Normalization: Not applicable

Normalization template: Not applicable

Noise and artifact removal: Not applicable

Volume censoring: Not applicable

Statistical modeling & inference

Model type and settings: Not applicable

Effect(s) tested: Not applicable

Specify type of analysis: ☐ Whole brain ☐ ROI-based ☐ Both

Statistic type for inference | Not applicable

(See [Eklund et al. 2016])

Correction | Not applicable

Models & analysis

n/a	Involved in the study
☑	☐ Functional and/or effective connectivity
☐	☑ Graph analysis
☑	☐ Multivariate modeling or predictive analysis

Functional and/or effective connectivity	Not applicable
Graph analysis	GraphPad Prism version 9.5.1
Multivariate modeling and predictive analysis	Not applicable

This checklist template is licensed under a Creative Commons Attribution 4.0 International License, which permits use, sharing, adaptation, distribution and reproduction in any medium or format, as long as you give appropriate credit to the original author(s) and the source, provide a link to the Creative Commons license, and indicate if changes were made. The images or other third party material in this article are included in the article's Creative Commons license, unless indicated otherwise in a credit line to the material. If material is not included in the article's Creative Commons license and your intended use is not permitted by statutory regulation or exceeds the permitted use, you will need to obtain permission directly from the copyright holder. To view a copy of this license, visit http://creativecommons.org/licenses/by/4.0/
